# High Plus Low Dose Radiation Strategy in Combination with TIGIT and PD1 Blockade to Promote Systemic Antitumor Responses

**DOI:** 10.3390/cancers14010221

**Published:** 2022-01-03

**Authors:** Hampartsoum B. Barsoumian, Duygu Sezen, Hari Menon, Ahmed I. Younes, Yun Hu, Kewen He, Nahum Puebla-Osorio, Mark Wasley, Ethan Hsu, Roshal R. Patel, Liangpeng Yang, Maria A. Cortez, James W. Welsh

**Affiliations:** 1 Department of Radiation Oncology, The University of Texas MD Anderson Cancer Center, Houston, TX 77030, USA; hbarsoumian@mdanderson.org (H.B.B.); dsezen@kuh.ku.edu.tr (D.S.); hmenon@uwhealth.org (H.M.); younesa20@ecu.edu (A.I.Y.); yhu9@mdanderson.org (Y.H.); hekewen@mail.sdu.edu.cn (K.H.); npuebla@mdanderson.org (N.P.-O.); mdwasley@mdanderson.org (M.W.); ehsu2@mdanderson.org (E.H.); roshal.r.patel@kp.org (R.R.P.); lpyang@mdanderson.org (L.Y.); macortez@mdanderson.org (M.A.C.); 2 Department of Radiation Oncology, Koc University School of Medicine, Istanbul 34450, Turkey; 3 Department of Human Oncology, University of Wisconsin Hospitals and Clinics, Madison, WI 53792, USA

**Keywords:** radiotherapy, immunotherapy, abscopal, TIGIT, lung cancer

## Abstract

**Simple Summary:**

This study combines a novel strategy of radiotherapy that utilizes high- and low- dose radiation with immune oncology agents (anti-TIGIT and anti-PD1 monoclonal antibodies) in order to overcome the inhibitory tumor stroma and battle tumors systemically. The findings from this work will impact how checkpoint inhibitors are delivered to maximize their efficacy.

**Abstract:**

Tumors deploy various immune-evasion mechanisms that create a suppressive environment and render effector T-cells exhausted and inactive. Therefore, a rational utilization of checkpoint inhibitors may alleviate exhaustion and may partially restore antitumor functions. However, in high-tumor-burden models, the checkpoint blockade fails to maintain optimal efficacy, and other interventions are necessary to overcome the inhibitory tumor stroma. One such strategy is the use of radiotherapy to reset the tumor microenvironment and maximize systemic antitumor outcomes. In this study, we propose the use of anti-PD1 and anti-TIGIT checkpoint inhibitors in conjunction with our novel RadScopal technique to battle highly metastatic lung adenocarcinoma tumors, bilaterally established in 129Sv/Ev mice, to mimic high-tumor-burden settings. The RadScopal approach is comprised of high-dose radiation directed at primary tumors with low-dose radiation delivered to secondary tumors to improve the outcomes of systemic immunotherapy. Indeed, the triple therapy with RadScopal + anti-TIGIT + anti-PD1 was able to prolong the survival of treated mice and halted the growth of both primary and secondary tumors. Lung metastasis counts were also significantly reduced. In addition, the low-dose radiation component reduced TIGIT receptor (PVR) expression by tumor-associated macrophages and dendritic cells in secondary tumors. Finally, low-dose radiation within triple therapy decreased the percentages of TIGIT^+^ exhausted T-cells and TIGIT^+^ regulatory T-cells. Together, our translational approach provides a new treatment alternative for cases refractory to other checkpoints and may bring immunotherapy into a new realm of systemic disease control.

## 1. Introduction

Despite current advances in cancer immunotherapy with checkpoint inhibitors such as anti-PD1, anti-PD-L1, and anti-CTLA-4, most patients do not realize the full benefit(s) due to intrinsic or acquired resistance. This is specifically a challenge in lung cancer, which is the second-most-common malignancy in the world. Finding ways to overcome immunotherapy resistance has become a challenging clinical question.

Radiotherapy (XRT) may provide a way to overcome innate resistance to immunotherapy. Traditionally, XRT has been used to control tumors locally by damaging their nucleic acid. More recently, we are beginning to understand that XRT helps release neo-antigens after cell death, upregulate MHC-I molecules [1], and prime T-cells. All of these functions may yield benefits in generating abscopal responses, which have eluded clinicians for decades. Our lab has previously shown that stereotactic XRT with selected I/O agents, such as anti-GITR or OX40 agonist, could promote abscopal responses in PD1-sensitive, as well as PD1-resistant murine solid tumors [1,2,3]. Others also reported the occurrence of abscopal responses in non-small-cell lung cancer (NSCLC) patients treated with radiotherapy and anti-CTLA-4, accompanied by the production of IFN-β cytokine [4]. The same treatment also expanded the T-cell receptor (TCR) repertoire in a murine breast carcinoma model [5]. More recently, we advanced our radiation technique to combine high-dose radiation (H-XRT) along with low-dose radiation (L-XRT) with an immunotherapy backbone to maximize systemic outcomes against secondary metastatic tumors, an approach we call the RadScopal technique [6]. The addition of L-XRT modulated the tumor microenvironment (TME) and its associated stroma to allow better infiltration of effector immune cells and enhanced the response to checkpoint inhibitors [6,7]. 

T-cell immunoreceptor with Ig and ITIM domains (TIGIT) was first described in 2009 as an immune checkpoint [8]. Over the past few years, it has emerged as a significant target in cancer immunotherapy. TIGIT has an extracellular type 1 transmembrane IgV domain and an intracellular immunoreceptor tyrosine-based domain. Human TIGIT shares 58% identity in its amino acid sequence with mouse TIGIT, and TIGIT’s cytoplasmic tail is identical in humans and mice [9]. TIGIT is expressed on both NK cells and T-cells, including CD4^+^ T-cells, CD8^+^ T-cells, and Tregs. While TIGIT expression is low in naive cells, it is usually increased by activation. 

TIGIT has three ligands, CD155 (poliovirus receptor or PVR), CD112 (Nectin-2), and CD113. TIGIT mainly interacts with CD155 expressed on dendritic cells (DCs), macrophages, B-cells, and various non-hematopoietic cells. CD112 has a broad expression in hematopoietic and non-hematopoietic tissues such as lung, bone marrow, and pancreas, while CD113 is limited to non-hematopoietic tissues. Expression of CD155 and CD112 can also be increased on tumor cells. TIGIT competes with the immune activator CD226 (DNAM-1) molecule for the same ligands: CD155 (PVR) and CD112 (Nectin-2 or PVRL2). CD226 is expressed on T-cells, NK cells, and monocytes. Contrary to TIGIT’s immunosuppressive effects, CD226 stimulates the cytotoxicity of T-cells and NK cells. CD96 is yet a third molecule expressed on T-cells and NK cells that binds to CD155 and mediates the immunosuppressive effects. When compared, TIGIT binds to CD155 with the highest affinity, followed by CD96 and then CD226. The TIGIT/CD226 pathway is similar to the CTLA-4/CD28 pathway, given that the inhibitory receptor has a higher binding affinity than the co-stimulatory receptor competing over the same ligand [10].

Multiple mechanisms have been described to explain TIGIT’s ability to suppress immune function. Following the ligation of TIGIT and PVR-expressing DCs, antigen presentation is decreased, and the production of proinflammatory cytokines such as interleukin (IL)-12 is reduced. On the other hand, anti-inflammatory cytokines such as IL-10 are increased; therefore, T-cell exhaustion and immunosuppression are intensified. Moreover, TIGIT initiates an inhibitory signal within T-cells and NK cells via its cytoplasmic tail and recruitment of SHP-1, ultimately inhibiting the PI3K and MAPK signaling cascades [11]. Blocking TIGIT by using an anti-TIGIT functional antibody is reported to enhance IFN-γ, IL-6, and TNF-α production [11].

TIGIT may impair T-cell proliferation even without the presence of antigen-presenting cells. TIGIT-deficient T-cells have been shown to have lower Foxp3^+^ expression. In addition, a subgroup of T-regulatory cells (Tregs) that are TIGIT^+^ frequently expresses an immunosuppressive gene signature including PD1 and CTLA-4, with a high suppressive profile [12]. Others have shown that T-cells that express both PD1 and TIGIT are highly exhausted and cannot exert effector functions [13]. Therefore, we hypothesized that adding H-XRT and L-XRT to anti-TIGIT plus anti-PD1 treatment may result in greater systemic antitumor outcomes. We hereby show that L-XRT can downregulate PVR expression on antigen-presenting cells (APCs), and the combination of RadScopal + anti-TIGIT + anti-PD1 may yield favorable local and systemic tumor control.

## 2. Materials and Methods

### 2.1. Cell Lines and Drugs 

The 344SQ parental (344SQ-P) lung adenocarcinoma cell line was used in this study. It is an aggressively growing cell line with a P53 mutation and KRAS hyperactivation (p53^R172HΔg/+^ K-ras^LA1/+^). Cells were cultured in RPMI-1640 medium supplemented with 100 U/mL penicillin, 100 mg/mL streptomycin, and 10% fetal bovine serum, then incubated at 37 °C in 5% CO_2_.

For drug preparation, both α-TIGIT (A3733F 4B1 mIgG1 D265A) and α-PD1 (clone 4H2-D265) blocking antibodies were obtained from Bristol-Myers Squibb and diluted in phosphate-buffered saline prior to intraperitoneal (i.p.) injections.

### 2.2. Mice

The experimental mice were 129Sv/Ev syngeneic male mice aged 8–12 wk. Mice were purchased from Taconic Biosciences and bred in house at the Experimental Radiation Oncology mouse colony facility at The University of Texas MD Anderson Cancer Center according to the Animal Care IACUC guidelines. 

### 2.3. Tumor Establishment and Treatments 

344SQ-P tumors were subcutaneously established in the right and left hind legs of 129Sv/Ev mice, 4 d apart, to establish primary (0.5 × 10^6^) and secondary (0.1 × 10^6^) tumors, respectively. Both primary and secondary tumors were measured twice per week using digital calipers. When primary tumors reached around 7 mm in diameter, they were locally irradiated using a cesium source (36Gy total, divided into 3 fractions of 12Gy each). For the RadScopal experiments, secondary tumors were also irradiated with L-XRT, 3 d after the last fraction of H-XRT to a dose of 2Gy total, divided over 2 fractions of 1Gy each. Anti-TIGIT and anti-PD1 were given on Days 5, 9, 12, 16, and 20 at 200 µg/i.p. injection. According to our protocol, mice were euthanized when the average tumor diameter reached 14 mm. Wherever specified, lungs were collected, stained with Bouin’s fixative solution, then enumerated for lung metastases. 

### 2.4. Tumor Processing and Flow Cytometry

Tumors were harvested, weighed, and processed to obtain single-cell suspensions. In brief, tumor tissues were dissociated and digested with 250 µg/mL of Liberase (Roche) and incubated for 30 min at 37 °C, while shaking at 105 rpm for proper digestion. Fetal bovine serum was then added to stop the reaction, and samples were filtered and washed with PBS + 2% FBS. The cell count per sample was performed, then samples were stained using fluorochrome-conjugated antibodies from BioLegend including: CD45 Pacific blue, cat# 103126; CD4 BV605, cat# 100451; CD8 PE, cat# 100707; CD49b APC, cat# 108910; Foxp3 Alexa488, cat# 126406; TIGIT PE-Cy7, cat# 142108; Gr1 BV510, cat# 108437; CD11b APC-fire750, cat# 101262; F4/80 Alexa700, cat# 123130; CD206 PercpCy5.5, cat# 141716. In alternate panels, a set of other antibodies was also used including: CD4 FITC, cat# 100406; CD8 PercpCy5.5, cat# 100734; Gr1 APC, cat# 108412; CD11c BV510, cat# 117337; and PVR (CD155) PE, cat# 132206. Samples were run on the Attune flow cytometer, and data were analyzed using Flow-Jo 10 software. 

### 2.5. Statistical Analysis

All statistical analyses were conducted with the GraphPad Prism 8 software. Mouse survival rates were analyzed using the Kaplan–Meier method and compared with log-rank tests. Tumor growth curves were compared using the two-way ANOVA method. Student *t*-tests were used to compare data between two individual groups where appropriate. The statistical analysis was considered significant at *p*-value ≤ 0.05. 

## 3. Results

### 3.1. High-Dose Stereotactic Radiation with TIGIT Blockade Improves Primary and Secondary Antitumor Efficacy

**Figure 1:**
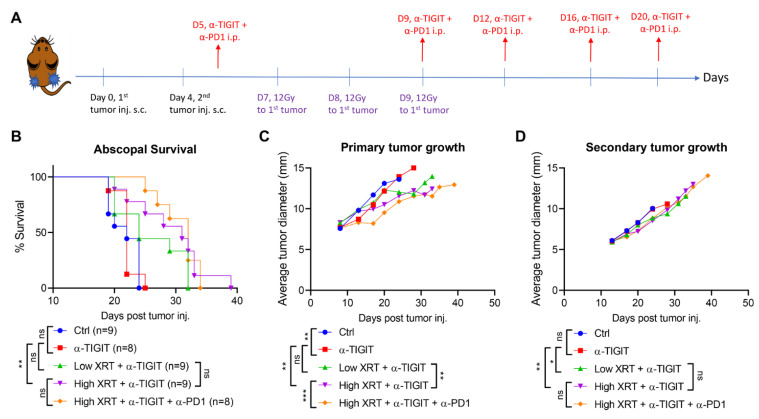
High-dose stereotactic radiation with TIGIT blockade improved primary and secondary antitumor efficacy. (**A**) Timeline and experimental design of the abscopal model. (**B**) Bilateral 344SQ-P tumors were established in 129Sv/Ev mice. Primary tumors were irradiated with H-XRT, while secondary tumors were left untreated. Systemic immunotherapy with α-TIGIT or α-TIGIT + α-PD1 was administered, as shown. Survival was monitored over 40 d observation period and graphed using the Kaplan–Meier method. (**C**) Primary and (**D**) secondary tumor growth curves are plotted over time, and different experimental groups were compared using two-way ANOVA. The experiment was repeated twice, and the data were pooled. *p* ≤ 0.05 was considered significant. * *p* ≤ 0.05, ** *p* ≤ 0.01, *** *p* ≤ 0.001, ns = not significant.

To assess the abscopal responses, bilateral 344SQ-P tumors were established in 129Sv/Ev mice on Days 0 and 4, respectively (Figure 1A). Primary tumors received a total radiation of 36Gy divided over three fractions of 12Gy each, while secondary tumors were left untreated. Anti-TIGIT and anti-PD1 monoclonal antibodies were delivered systemically on Days 5, 9, 12, 16, and 20 (Figure 1A). H-XRT + α-TIGIT with or without α-PD1 scored the longest survival over the 40 d observation period (Figure 1B) (H-XRT + α-TIGIT median survival = 31 d). Monotherapy with α-TIGIT alone (median survival 22 d) did not show a survival benefit, and all mice expired in a similar tempo to the control group (Ctrl, median survival 22 d). In another experimental group, primary tumors were treated with L-XRT (2Gy total, divided over two fractions of 1Gy each) and systemic α-TIGIT; however, H-XRT was found superior to L-XRT in improving overall survival when compared to α-TIGIT alone (α-TIGIT vs. L-XRT + α-TIGIT, *p* = 0.0861; α-TIGIT vs. H-XRT + α-TIGIT, *p* = 0.0058). Tumor growth curves were also monitored for both primary (Figure 1C) and secondary tumors (Figure 1D). The H-XRT + α-TIGIT + α-PD1 efficacy was more accentuated in primary tumors than secondary. Anti-TIGIT monotherapy slowed down primary tumor growth when compared to Ctrl (*p* = 0.0034), but that was not observed in secondary tumors. Adding H-XRT to α-TIGIT led to retardation in tumor growth of both primary (α-TIGIT vs. H-XRT + α-TIGIT, *p* = 0.0032) and secondary/abscopal tumors (α-TIGIT vs. H-XRT + α-TIGIT, *p* = 0.0011), while adding α-PD1 to H-XRT + α-TIGIT did not magnify the abscopal response (*p* = 0.4517).

### 3.2. RadScopal Approach with Anti-TIGIT Plus Anti-PD1 Immunotherapy Had a High Impact on Secondary Tumors 

**Figure 2:**
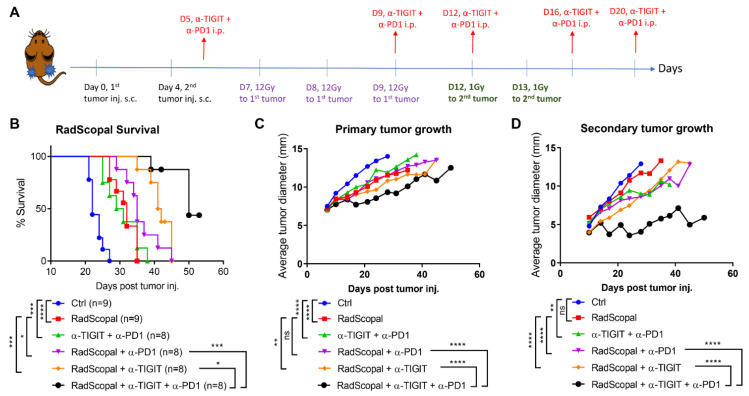
Triple therapy with RadScopal + α-TIGIT + α-PD1 significantly hampered secondary tumors’ growth. (**A**) Timeline and experimental design of the RadScopal model. (**B**) 344SQ-P tumors were established bilaterally in 129Sv/Ev mice similarly to Figure 1, with the exception that secondary tumors were irradiated with L-XRT (1Gyx2). Survival was monitored over 53 d and graphed using the Kaplan–Meier method. (**C**) Primary and (**D**) secondary tumor growth curves are plotted over time, and experimental groups were compared using two-way ANOVA analysis. The experiment was repeated twice, and the data were pooled. *p* ≤ 0.05 was considered significant. * *p* ≤ 0.05, ** *p* ≤ 0.01, *** *p* ≤ 0.001, **** *p* ≤ 0.0001.

In order to amplify the abscopal response and help reach its full potential, the same experimental design was used from Figure 1A, except that the secondary tumors were treated with a non-ablative immunostimulatory L-XRT dose, as described before [6] (Figure 2A). The RadScopal technique harnesses the benefits of both H-XRT and L-XRT in combination with checkpoint inhibitors to maximize the control of secondary/metastatic tumors and overcome the inhibitory stroma. In this set of experiments, the RadScopal group’s median survival was 32 d compared to the control of 22 d (Figure 2B), and α-TIGIT + α-PD1 median survival was 30 d; however, when RadScopal was combined with α-TIGIT and α-PD1 as a triple therapy, the median survival observed was 50 d. Adding L-XRT to secondary tumors within the RadScopal frame significantly improved the survival of α-PD1 (RadScopal vs. RadScopal + α-PD1, *p* = 0.05) and that of α-TIGIT treatment (RadScopal vs. RadScopal + α-TIGIT, *p* = 0.0002). Moreover, the triple therapy significantly abated the growth of primary (Figure 2C) and secondary tumors (Figure 2D) with a more pronounced influence on secondary tumors as compared to RadScopal + α-PD1 (*p* ˂ 0.0001) or RadScopal + α-TIGIT (*p* ˂ 0.0001) dual therapies. In addition to that, the triple therapy group reduced lung metastases’ counts in this high-tumor-burden and aggressively spreading 344SQ-P model when compared to RadScopal alone (*p* = 0.0297) or immunotherapy alone (α-TIGIT + α-PD1, *p* = 0.0477) (Figure S1A). Importantly, the triple therapy efficacy was associated with effector immune memory generation for both CD4^+^ and CD8^+^ T-cell compartments (Figure S1B). The level of effector memory observed was highly comparable to our conventional RadScopal treatment with the α-CTLA-4 + α-PD1 backbone, which we included as a positive control. 

### 3.3. Low-Dose Radiation Reduces the TIGIT Receptor’s Expression in the TME

**Figure 3:**
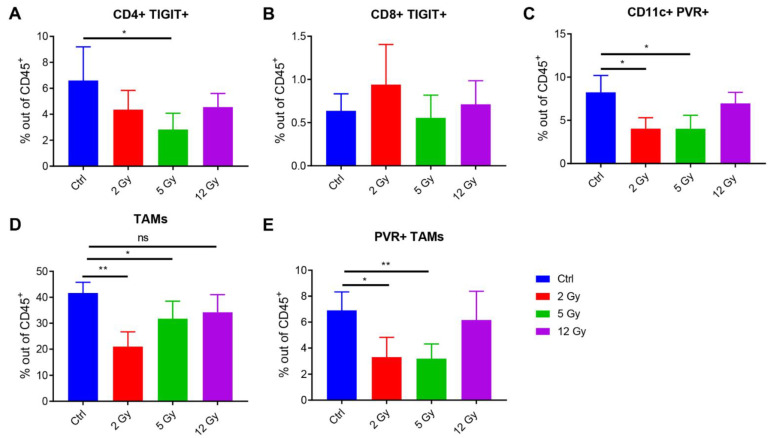
Lower non-ablative doses of radiation reduced PVR expression in the TME. (**A**–**E**) Flow cytometric analysis of TILs harvested 48 h after radiation (n = 4/group). (**A**,**B**) TIGIT expression was evaluated by CD4 and CD8 T-cells after gating on CD45^+^ lymphocytes. (**C**) PVR percentages were evaluated in CD11c^+^ dendritic cells after gating on CD45^+^ leukocytes. (**D**,**E**) PVR levels were also evaluated on Gr1^intermediate^ CD11b^+^ tumor-associated macrophages (TAMs). Student *t*-tests were conducted to compare the statistical significance between two groups with *p* ≤ 0.05 considered significant. * *p* ≤ 0.05, ** *p* ≤ 0.01.

In an effort to understand how low doses of radiation may impact the TIGIT/PVR axis, we conducted flow cytometric analysis on tumors harvested 48 h after different doses of radiation (low dose of 2Gy, intermediate dose of 5Gy, and high dose of 12Gy). XRT in general reduced the percentages of CD4^+^ TIGIT^+^ T-cells and reached significance with the 5Gy dose, *p* = 0.0391, vs. Ctrl (Figure 3A). The same trend was not observed for CD8^+^ TIGIT^+^ T-cells (Figure 3B). On the other hand, the PVR expression on CD11c^+^ dendritic cells (Figure 3C) and tumor-associated macrophages (TAMs) (Figure 3D,E) was significantly reduced with the low and intermediate XRT doses, but not with the higher 12Gy dose ((DCs’ panel: 2Gy vs. Ctrl *p* = 0.0113, 5Gy vs. Ctrl *p* = 0.0150); (PVR^+^ TAMs’ panel: 2Gy vs. Ctrl *p* = 0.0135, 5Gy vs. Ctrl *p* = 0.0064)). To our knowledge, this is the first time low-dose radiation has been reported to reduce PVR-expressing immune populations in the TME. This novel observation is important to reduce exhaustion and complement anti-TIGIT treatment efficacy, especially at secondary tumor sites treated with L-XRT.

### 3.4. RadScopal Treatment Reduces TIGIT-Expressing T-Cell Populations 

**Figure 4:**
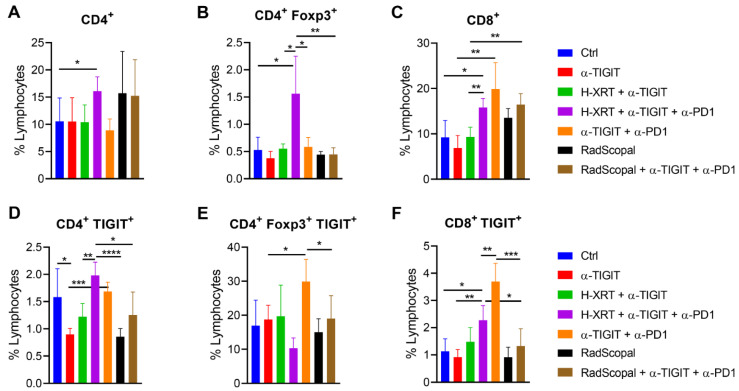
RadScopal treatment reduced TIGIT-expressing T-cell populations. (**A**–**F**) The RadScopal model was established as in Figure 2A. On Day 20, secondary tumors were harvested and processed for flow cytometric analysis. (**A**–**C**) Cells were first gated on lymphocytes, followed by CD45, and then on CD4 vs. CD8, or CD4^+^ Foxp3^+^ Tregs. (**D**,**F**) Cells were gated on lymphocytes, then CD45, then on either CD4^+^ TIGIT^+^ or CD8^+^ TIGIT^+^ populations. (**E**) Cells were gated on lymphocytes, then CD45, then CD4^+^ Foxp3^+^ Tregs, then TIGIT^+^ Tregs. Student *t*-tests were used to compare the statistical significance between two groups with *p* ≤ 0.05 considered significant. * *p* ≤ 0.05, ** *p* ≤ 0.01, *** *p* ≤ 0.001, **** *p* ≤ 0.0001.

To determine if L-XRT is capable of reducing T-cell exhaustion in secondary tumors, we established the two-tumor model with 344SQ-P in 129Sv/Ev mice, similar to the experimental design in Figure 2A. H-XRT was delivered to primary tumors on Days 7, 8, and 9, while L-XRT was delivered to secondary tumors on Days 12 and 13. On Day 20, secondary tumors were harvested, processed into single-cell suspensions, and stained with cell-surface and intracellular markers to conduct flow cytometry. The results showed that the H-XRT + α-TIGIT + α-PD1 group was capable of increasing the percentages of total CD4^+^ T-cells (Figure 4A), CD4^+^ Foxp3^+^ Tregs (Figure 4B), as well as total CD8^+^ T-cells (Figure 4C) at the unirradiated secondary tumor site compared to the Ctrl group (*p* = 0.05, *p* = 0.0251, *p* = 0.0111, respectively). On the other hand, there was a strong trend with RadScopal treatment with or without immunotherapy to increase CD4^+^ T-cells (Figure 4A) that were not Foxp3^+^ Tregs for most (Figure 4B). The effect of α-PD1 was more pronounced in the CD8^+^ compartment (Figure 4C) (α-TIGIT vs. α-TIGIT + α-PD1, *p* = 0.0071; H-XRT + α-TIGIT vs. H-XRT + α-TIGIT + α-PD1, *p* = 0.0022). However, adding α-PD1 to H-XRT + α-TIGIT or even to α-TIGIT alone increased the percentages of CD4^+^ TIGIT^+^ and CD8^+^ TIGIT^+^ T-cells ((Figure 4D, H-XRT + α-TIGIT vs. H-XRT + α-TIGIT + α-PD1, *p* = 0.0024; α-TIGIT vs. α-TIGIT + α-PD1, *p* = 0.0007); (Figure 4E, α-TIGIT vs. α-TIGIT + α-PD1, *p* = 0.0284); (Figure 4F, H-XRT + α-TIGIT vs. H-XRT + α-TIGIT + α-PD1, *p* = 0.0621; α-TIGIT vs. α-TIGIT + α-PD1, *p* = 0.0003)). This indicated that blocking PD1 led to upregulation of TIGIT. L-XRT in turn was capable of significantly diminishing CD4^+^ TIGIT^+^, Foxp3^+^ TIGIT^+^, and CD8^+^ TIGIT^+^ populations in both the RadScopal-only group and the RadScopal + α-TIGIT + α-PD1 group ((Figure 4D, H-XRT + α-TIGIT + α-PD1 vs. RadScopal + α-TIGIT + α-PD1, *p* = 0.0103); (Figure 4E, α-TIGIT + α-PD1 vs. RadScopal + α-TIGIT + α-PD1, *p* = 0.0454); (Figure 4F, H-XRT + α-TIGIT + α-PD1 vs. RadScopal + α-TIGIT + α-PD1, *p* = 0.0268)).

## 4. Discussion

Our study aimed to reveal that adding H-XRT and L-XRT to anti-TIGIT treatment may result in more outstanding in vivo antitumor outcomes with the anti-PD1 backbone, and this combination may produce better local and systemic tumor control with enhanced survival.

The effects of TIGIT are better known on T-cells compared to other immune cell groups. TIGIT was shown to be upregulated on CD8^+^ T and CD4^+^ cells with activation. It indirectly inhibits T-cell responses by binding CD155 on DCs and macrophages, restraining APCs’ maturation, provoking the immunosuppressive cytokine IL-10, and suppressing IL-12 Th1 cytokine [8]. TIGIT plays a major role in Tregs as well. One study reported that the function of TIGIT in Tregs is more important in dampening antitumor immune responses compared to its function in effector CD8^+^ T-cells [12]. TIGIT can also upregulate the expression of the chemokine CCL4 and the chemokine receptor CCR8, which help with Treg migration and retention in tumor tissue [12]. Recent reports showed that Treg cells have diverse subtypes with different phenotypes and specific functions. In this context, Joller et al. [14] evaluated whether TIGIT has a functional role in these cells and showed that TIGIT contributes to the selective Treg-cell-mediated suppression of Th1 and Th17 cells, but not Th2 cell responses. Therefore, when compared to TIGIT^−^ Treg cells, TIGIT^+^ Treg cells are a highly suppressive subset, with higher Foxp3 and IL-10 expression. Our results showed that the triple therapy with RadScopal + α-TIGIT + α-PD1 was able to reduce the percentages of CD4^+^ Foxp3^+^ TIGIT^+^ Tregs, as well as CD8^+^ TIGIT^+^ T-cells.

The synergistic effects of radiation and immunotherapy are well established with recent preclinical and clinical research. However, the literature on combining radiation with TIGIT blockade is scarce. Grapin et al. [15] evaluated how the radiation dose per fraction could modulate the immune system for schedules with similar biologically effective doses. This was also one of the first studies combining XRT and anti-TIGIT with promising results. For this purpose, mice bearing CT26 colon tumors were irradiated with 2Gyx18, 8Gyx3, and 16.4Gyx1 radiation schemes that had the same biologically effective dose. Each fractionation scheme produced different lymphoid and myeloid responses and several modulations of PD-L1 and TIGIT expression. While 8Gyx3 and 16.4Gyx1 caused a lymphoid response with induced CD8^+^ T-cells and Tregs, the 2Gyx18 regimen led to a myeloid response through M2 TAMs. In the same study, they also showed that TIGIT expression by CD8^+^ T-cells was increased with 8Gyx3, while it was reduced by 2Gyx18 (*p* < 0.05). However, anti-TIGIT produced a significant antitumor effect only when combined with anti-PD-L1 and the 8Gyx3 scheme. These findings confirm our data that H-XRT (12Gyx3) upregulates TIGIT^+^ T-cells, and abscopal responses were mostly observed when combining H-XRT + anti-TIGIT + anti-PD1. To further boost the efficacy of the immunotherapy agents used, we devised the RadScopal technique that incorporates L-XRT treatment at secondary tumor sites to overcome the inhibitory stroma and maximize systemic outcomes. Indeed, L-XRT dampened T-cell exhaustion by reducing the percentages of TIGIT-expressing T-cells, as shown by flow phenotyping. Moreover, L-XRT reduced PVR-expressing APCs in the TME of treated tumors, which shifts the balance towards costimulation rather than immune suppression.

Although we did not report data in regard to NK cells in this study, we have previously shown that L-XRT significantly enhances the infiltration of NK cells into the TME of secondary tumors [6]. Others observed that TIGIT expression on tumor-infiltrating NK cells was associated with tumor progression in preclinical models [16]. Moreover, TIGIT deficiency in NK cells alone was sufficient to delay tumor growth independently of the adaptive immune system. In the same study, while TIGIT^+^ T-cells were mostly PD1^+^ or CTLA-4^+^ in all tumor-bearing models, TIGIT^+^ NK cells were mostly both PD1^–^ and CTLA-4^–^. Other checkpoint inhibitors under current scrutiny, such as LAG-3 and TIM-3, remain to be explored in combination with anti-TIGIT and XRT for systemic antitumor efficacy.

In the context of designing clinical trials with anti-TIGIT agents, certain biomarkers (NCR1, IFN-γ, GranzymeA, GranzymeB, and CD226 costimulatory molecule) can serve as indirect readouts in tumors and the blood of patients to measure the successful blockade of the TIGIT-PVR axis and can be coupled with clinical response outcomes [17,18]. A recent study showed that the percentage of CD8 T-cells that were TIGIT^+^ increased in gastric cancer patients compared to healthy individuals [19]. These cells exhibited functional exhaustion and reduced metabolic activity. However, anti-TIGIT treatment results in humans are currently limited or even unknown. To date, clinical trials with anti-TIGIT as a monotherapy or with other immunotherapies are under investigation. In a phase III trial, Tiragolumab (an anti-TIGIT antibody) and Atezolizumab (an anti-PD-L1 antibody) will be compared with placebo plus Atezolizumab in patients with previously untreated locally advanced, unresectable, or metastatic PD-L1-selected non-small cell lung cancer (NCT04294810). In a second phase III trial, in which patient recruitment has not been initiated yet, Atezolizumab plus Carboplatin and Etoposide with or without Tiragolumab will be tested in patients with untreated extensive-stage small-cell lung cancer (NCT04256421). So far, there are no clinical trials that utilize radiation with anti-TIGIT combination. Future trials are warranted to help understand whether TIGIT is a potential add-on to next-generation immunotherapies, especially with radiation treatment.

## 5. Conclusions

The triple combination “RadScopal treatment and α-TIGIT and α-PD1” suppressed the growth of primary and secondary tumors in a lung adenocarcinoma murine model. The treatment primarily reduced the exhaustion of T-cells and generated effector immune memory.

## Data Availability

All data are contained within the article or Supplementary Material.

## Supplementary Materials

The following are available online at https://www.mdpi.com/article/10.3390/cancers14010221/s1, Figure S1: RadScopal + α-TIGIT + α-PD1 triple therapy reduces lung metastases and generates immune memory.
