# Supplementary material for: High Plus Low Dose Radiation Strategy in Combination with TIGIT and PD1 Blockade to Promote Systemic Antitumor Responses
Source: Cancers (Basel). 2022 Jan 3;14(1):221. doi: 10.3390/cancers14010221 (PMC8750272; doi:10.3390/cancers14010221)
Supplement: Supplementary file 1 [file cancers-14-00221-s001.zip › cancers-1517495-supplementary.pdf]

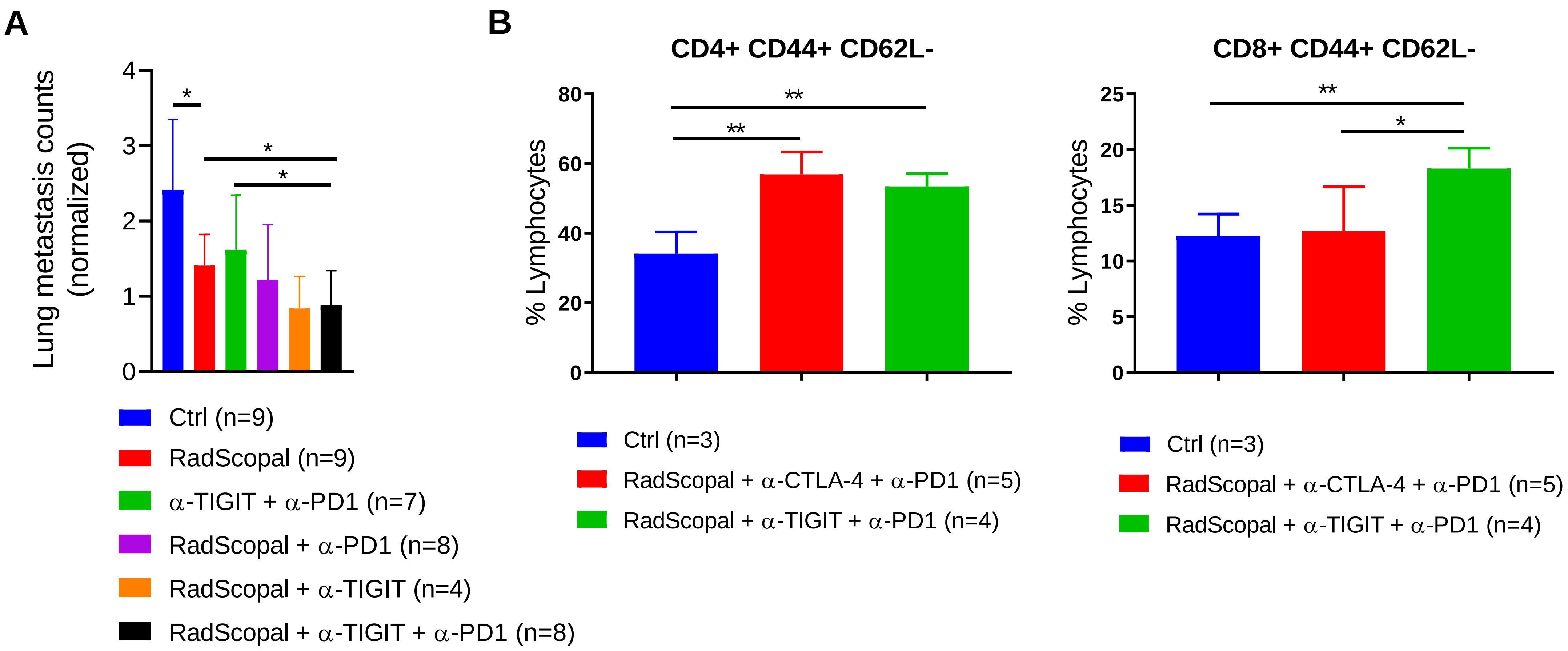

Supplementary Figure S1. RadScopal + α-TIGIT + α-PD1 triple therapy reduces lung metastases and generates immune memory. (**A**) Lungs were collected from different experimental groups as mice expired, and were stained with Bouin's fixative solution, then lung lesions were enumerated, divided by the day of sacrifice, and reported accordingly. (**B**) On day 40, spleens were harvested from the triple combination therapy group and compared to tumor-bearing control and RadScopal + α-CTLA-4 + α-PD1 groups for effector memory phenotyping of T-cells using flow cytometry. Cells were gated on lymphocytes then on either CD4 or CD8 populations, followed by $CD44^+$ and $CD62L^-$. Data was reported as mean ± SD. Groups were compared using Student t-tests, with $p \leq 0.05$ considered to be statistically significant. * $p \leq 0.05$, ** $p \leq 0.01$.
